# Student-Teacher Relationships As a Protective Factor for School Adjustment during the Transition from Middle to High School

**DOI:** 10.3389/fpsyg.2016.01988

**Published:** 2016-12-23

**Authors:** Claudio Longobardi, Laura E. Prino, Davide Marengo, Michele Settanni

**Affiliations:** Department of Psychology, University of TurinTurin, Italy

**Keywords:** student-teacher relationship, school transition, longitudinal study, academic achievement, middle school, high school

## Abstract

A robust body of research has identified school transitions during adolescence, and in particular the transition from middle to high school, as one of the riskiest phases for school failure, being characterized by significant social, emotional and behavioral changes. This transition is critical even with respect to academic achievement: in Italy, the highest frequency of school dropout can be observed in the 9th and 10th grades, partly as a consequence of poor adjustment to the new school context. The impact of students' relationships with their teachers may be particularly relevant during critical developmental periods. Indeed, student-teacher relationships have been widely recognized as protective factors in school adjustment and, in case of negative relationships, also as a factor that increases the risk of maladjustment. Positive and affective student-teacher relationships may play an important role in students' adaptation to the school environment, favoring both academic achievement and adaptive behaviors. The aim of this study was to investigate the effects of the quality of teacher-student relationships, as perceived by pupils, on academic achievement, and problem and prosocial behaviors during the relevant school transition. The sample consisted of 122 students (55% female). We employed a self-report questionnaire to collect information on: demographic characteristics, quality of the relationship with teachers, problem and prosocial behaviors, and academic achievement. Students filled in the questionnaires twice: once during the 8th grade and 1 year later, during their first year of high school (9th grade). Regression analyses indicated that both average and varying levels of closeness with teachers significantly predicted changes in academic achievement: A perceived increase in closeness in 9th grade, as well as a higher mean closeness level, was associated with an increase in academic achievement. In turn, an increase in the level of perceived conflict with teachers significantly predicted an increase in conduct problems and hyperactive behaviors. This study supports the significance of student-teacher relationships as a protective factor during students' transition to high-school. Our findings also highlight the importance of relationship quality in preventing students' risk of school failure.

## Introduction

It is well established that one of the most demanding phases for children is that of school transition, especially the one from middle to high school (Gazelle and Druhen, 2009; Shell et al., 2014). Entering a new learning context requires students to adapt to harder tasks and to achieve different goals, therefore placing great pressure on their emotional well-being (Scalera and Alivernini, 2010).

The first empirical research studies on this theme date back to the end of the 1900s in the United States and, since the 1980s, a substantial line of international and inter-disciplinary research interested has developed (Neild, 2009). In 2000, the International Journal of Educational Research (Galton et al., 2000) presented a monographic issue on normative school transitions, discussing literature from an increasingly large number of geographic areas (Darmody, 2008; Cueto et al., 2010; Jindal-Snape, 2010), and in particular Germany (Van Ophuysen, 2009), Switzerland (Neuenschwander and Garrett, 2008), and Belgium (Dang Kim and Pelleriaux, 2006). Unlike other countries, in Italy research on school transitions is scarce (Scalera and Alivernini, 2010). Among the few existing studies are those by Pombeni and D'Angelo (1994) on the theme of motivation in learning and scholastic orientation, the study by Scalera and Alivernini (2010) on the transition to high school, as well as researches on the transition from primary to middle school by Zanobini and Usai (2002) and Tomada et al. (2005).

The transition to high school has been described as being the most critical when compared to other school transitions (Southern Regional Education Board, 2002; Barber and Olsen, 2004; Scalera and Alivernini, 2010; Ellerbrock and Kiefer, 2013; Roybal et al., 2014), especially because of its high dropout and failure rate (National Center for Education Statistics, 2008). In Italy, for example, 20.3% of new enrolments in the first year of high school fail (ISTAT, 2011). Such high failure rates highlight the importance of risk assessment for teachers, educators, school psychologists, and policy makers. In light of these considerations, the present study aims to investigate student-teacher relationships (STRs) as factor in promoting students' psychosocial adjustment during the transition from middle to high school. More in detail, the aim of this work is to assess, in the transition from middle school to high school, whether there is a link between the quality of the relationship with teachers as perceived by their students, academic achievement and problem or prosocial behavior. We hypothesize that STR quality may be an important emotional resource for school transitions, favoring the scholastic adjustment of the students (e.g., limiting problems behavior and promoting academic achievement).

### Transitioning to high-school: risk and protective factors

There are numerous factors that make it difficult to adapt to high school, chiefly the fact that students are simultaneously entering adolescence, which involves a complex redefinition of their personalities (Erikson, 1968; Blos, 1988), alongside difficulties in maintaining positive emotional well-being (Akos, 2002; Frey et al., 2009; Neild, 2009). Risk factors include, but are not limited to: larger, more chaotic rooms; school organization marked by a greater deal of bureaucracy; and a heavier workload, requiring increased cognitive effort on behalf of students (Akos and Galassi, 2004; Scalera and Alivernini, 2010; Eccles and Roeser, 2011; Waters et al., 2012). An additional relevant factor is the change that occurs in students' relationships with teachers and peers. New teachers tend to be perceived as cold, impersonal, and unreceptive to their developmental needs. Furthermore, students face relevant changes in their friendship networks (Cushman and Rogers, 2008; National Middle School Association, 2010; Scalera and Alivernini, 2010; Eccles and Roeser, 2011). Students have to reconsider their position in a new peer group, conscious of losing the security they have developed in a familiar classroom. For particularly anxious children, fitting into a new peer group may be a problem and could call for specific interventions (Gazelle, 2006; Oh et al., 2008).

The transition causes a series of changes, making it necessary for students to reorganize their social lives, and requiring them to cope with the new adaptation and development tasks. In the new school environment, students may perceive a lack of support from teachers and peers, and face difficulties in regulating their behavior. A reduction of emotional support in the transition to high school may result in a significant increase in the number of students who suffer from some form of exclusion (Avant et al., 2011). More specifically, if students needing more protection than others in this transition phase lack emotional support, their process of integration is hindered and they may also be exposed to experiences of victimization (Gazelle, 2008). Conversely, many studies have documented that a warm classroom climate, fostered by the social support of teachers, parents and peers, promotes lower conduct problems (Wang and Eccles, 2012). Furthermore, the transition to a new class may also provide students with positive opportunities to establish more satisfying and gratifying relationships with peers (Li and Lerner, 2011), especially for students with a previous history of victimization (Gazelle et al., 2005).

Alongside peer support, teachers' willingness and ability to support their students during developmental transitions remains a crucial factor in favoring their adaptation to the new environment. Students who experience some form of support from their teachers show increased academic commitment and motivation to learn (Fraire et al., 2013), as well as higher positive social and emotional well-being. In spite of this, it is often the case that teachers' management style, in an effort to maintain discipline and control over school activities, may compromise students' successful adaptation to the new requirements (Eccles and Roeser, 2011). In particular, this happens in cases where great importance is given to formal assessment. Students with low marks may perceive their teachers as unsupportive and ill-disposed toward them. At the same time, schools as institutions will be perceived as an unpleasant, pointless and, at times, hostile places (Bru et al., 2010).

In sum, it can certainly be argued that the teaching ability and relational skills of teachers are important to stimulate and promote students' motivation to learn while at school (Wentzel, 1998; Chen, 2008). Teachers who are able to regulate classroom activities, while also highlighting students' progresses and achievements, significantly help their students during the transition and adaptation to the new school environment (Ryan and Deci, 2000).

### Student-teacher relationship and behavioral problems

Alongside being a place for learning, classrooms are living environments in which many significant interpersonal relationships are developed. In this setting, teachers are central, and the quality of their relationships with students is fundamental to many aspects of school life. Children experiencing positive relationships with their teacher develop interest in school activities, are more motivated and willing to learn (Baumeister and Leary, 1995; Wentzel et al., 2010; Prino et al., 2016), and show higher academic achievement (Hughes, 2011; Pasta et al., 2013). Additionally, a positive link exists between the emotional support provided by student-teacher interactions and students' development of relational and social skills (Pianta et al., 2008b). Therefore, students' perception of emotional support is essential for their correct development, favoring learning and the creation of a wider network of friends (Pianta et al., 2008a).

Studies investigating the role of STRs in promoting students' well-being and academic achievement in the perspective of attachment theory, have shown that teachers which act as a “secure base”—that is, being available, responsive and accepting of students' needs—improve their students' commitment (Hughes et al., 2008; Myers and Pianta, 2008; Gastaldi et al., 2015), competence (Baker, 2006), and favor the development of their learning interests (Hughes et al., 2008; Quaglia et al., 2013). Low-conflict relationships with teachers favor an increase in positive classroom climate and students' perceived teacher support, and a decrease in students' negative experiences (Hamre et al., 2008; O'Connor, 2010). Teachers who share a warm relationship with their students tend to develop a positive sense of community in the classroom, as well as to promote cooperation among students by favoring their sharing of skills and ideas. Students seem to interiorize the interactions they have with their teachers and reproduce them with their classmates. In other words, if teachers behave in a consistent, accessible manner with their students, the latter tend to behave in the same way with their classmates (Mikami et al., 2011; Settanni et al., 2015). Conversely, children who are more isolated tend to relate less with their teachers (Wu et al., 2010). Similarly, aggressive children and those with low interest for school activities tend to relate very little with their teachers (Gest and Rodkin, 2011). The quality of friendships between peers is often compromised in children that show aggressiveness or lack of respect for others (O'Connor, 2010). Therefore, improving children's relationships with their teachers and peers is essential, not just to promote motivation and commitment, and to support the resilience of vulnerable students, but also to avoid or interrupt behaviors that threaten positive psychological growth (Bronfenbrenner and Morris, 2006).

### Current study

In Italy, schools are organized in a way that continuity of the class group is maintained within school cycles. Each class is formed by a group of students who normally stay together for the whole length of the school cycle, that is, for three consecutive years in middle school, and 5 years in high schools. Within-cycle changes in the class composition are rare, as there is much less mobility in Italy as compared, for example, to the United States or the United Kingdom. Teachers are also generally quite stable in the class: in some cases, they teach the same group of students for the whole school cycle. The continuity of the class group is significant in psychological terms, since with the passing of time students develop a sense of belonging, share ideas and visions of schooling, teaching, and learning. Moreover, and differently from other countries, no curricular flexibility is allowed to the students in Italian middle and high schools. By the end of middle school, at the age of 13–14 years, students are required to choose the track they intend to follow the next 5 years of high school.

The aim of this study is to investigate the effects of the quality of teacher-student relationships, as perceived by students, on their academic achievement and problem and prosocial behaviors during this important school transition. Regarding behaviors, we consider problematic ones as possible risk factors for school dropout. Indeed, as posited by many authors, dropping out of school is the culmination of cumulative risk factors over time, including poor academic achievement, school disengagement, and a variety of childhood behavior problems. In this study we examine whether students' individual relationship with their teachers during the transition from 8th to 9th grade predicts a change in academic achievement and in other behavioral difficulties related to the risk of school failure. Based on previous considerations about the protective role of student-teacher relationship quality in improving students' academic success and psychosocial adjustment, we hypothesize that positive transition-related changes in STR quality will have a positive impact on students' academic achievement and behavioral outcomes.

## Methods

### Participants and procedure

Sample consists of 181 Italian 8th grade students recruited from different middle schools in Northern Italy. After 1 year, participants were contacted in their new schools. However 59 participants were lost to follow-up given that some of the new schools did not give consent for the research to continue. The final sample consists of 122 students (of which 55% female). We employed a self-report questionnaire to collect information regarding demographic characteristics (age, gender), quality of relationship with teachers (using the Student Perception of Affective Relationships with Teacher Scale—SPARTS, Koomen and Jellesma, 2015), problematic and prosocial behavior (Strenghts and Difficulties Questionnaire, SDQ, Goodman, 1997) and academic achievement (as the average grade across all the school subjects).

Students filled in the questionnaires twice: first during the 8th grade and then 1 year later during their first year of high school (9th grade).

### Ethical considerations

School principals gave their consent for the participation of both teachers and students in our study. Individual informed consent to take part in the research was also collected from teachers, children and their parents, along with written consent describing the nature and objective of the study according to the ethical code of the Italian Association for Psychology (AIP). The consent stated that data confidentiality would be assured and that participation was voluntary. For the pupils, both parents were asked to sign the consent form in order to have their child participate in our study. The study was approved by the IRB of the University of Turin (approval number: 42345).

### Instruments

After collecting data about students' age and gender, both students and teachers were asked to fill in a questionnaire including the following instruments.

#### Strengths and difficulties questionnaire (SDQ)

Teachers were asked to fill in the SDQ (Goodman, 1997, 1999; for the Italian validation, see Tobia et al., 2011), which is a brief behavioral screening questionnaire for children and adolescents aged 3–16. It consists of 25 items investigating 5 different dimensions: Emotional symptoms, Conduct problems, Hyperactivity/inattention, Peer relationship problems and Prosocial behavior. Teachers evaluated the degree to which each item (such as: “Considerate of other people's feelings”; “Has at least one good friend”) described the student, using a 3-point Likert scale (0: Not true, 1: A little true, 2: Certainly true). Subscales' Cronbach's α for this study ranged from 0.65 to 0.86; the average α was 0.74.

#### Academic achievement

Teachers were asked to report the average grade obtained by each student across all the school subjects. Each school subject was graded on a 1–10 scale.

#### Student perception of affective relationship with teacher scale (SPARTS)

We examined students' perceptions of the student–teacher relationship quality using the Student Perception of Affective Relationship with Teacher Scale (SPARTS; Koomen and Jellesma, 2015). It consists of 25 items investigating three dimensions, namely Closeness, Conflict, and Negative expectations. The Closeness scale (8 items) assesses the students' positive feelings toward and reliance on their teacher (e.g., “I feel most at ease when my teacher is near”). Conflict dimension (10 items) measures the pupils' perception of the extent of negative behavior, and attitudes experimented with their teacher (e.g., “I guess my teacher gets tired of me in class”). Negative expectations scale (7 items) measures the lack of confidence in teacher's responsiveness and availability, (e.g., “I wish my teacher knew me better”). Children evaluated the extent to which they believed each of the 25 statements applied to their relationship with the teacher on a 5-point response scale, ranging from 1 (“no, that is not true”), to 5 (“yes, that is true”). Cronbach's alphas for this study were adequate, ranging from 0.66 to 0.84, the average α was 0.77.

### Data analysis

As a first step, study measures were inspected for univariate outliers using *Z-scores* (−3.29 < *Z* < 3.29). Analyses revealed presence of outliers on the SDQ subscales (T2) assessing emotional symptoms (3), conduct problems (1) and peer-relationship problems (1). Comparison of mean scores of these variables with corresponding means after removing outliers showed that none of these outliers significantly influenced mean scores on the variables at a nominal alpha level of 0.05. Consequently, univariate outliers were retained within further analyses (Pallant, 2001).

Then, descriptive statistics (mean, standard deviation, range) were computed on the study variables, both in the overall sample and by gender group. Independent samples *t*-tests were performed to investigate significance of gender differences on study measures. In order to investigate the significance of mean changes over time on the study measures, a set of paired-samples *t*-tests were performed in the overall sample. A measure of effect size (Cohen's *d*) was used to convey the size of difference in study measures between the two time points.

In order to investigate univariate relationships between study measures, Pearson's correlation coefficients were computed on measures as assessed at T1.

Descriptive statistics (mean, standard deviation, range) were computed on the study variables. In order to investigate the significance of mean changes over time on the study measures, a set of paired-samples *t*-tests were additionally performed.

A set of multiple linear regression models was utilized to investigate the link between students' relationship quality with teachers and 1-year follow-up measures of achievement and emotional and behavioral difficulties. Potential collinearity among IVs was controlled by mean-centering the variables (Aiken and West, 1991). Predictors were then entered in the regressions in the form of both time-averaged levels and change scores. More in detail, t1 and t2 measures of achievement and emotional and behavioral difficulties (X1, X2) were entered into the analyses both as an average level (X1 + X2)/2 and as a difference (X2 − X1). We choose to use this specific parameterization approach as to determine in the analyses the presence of participants who have either stably low or high scores on the predictor variables included in the models (Labouvie et al., 1991). Thus, we investigated the associations between mean and difference scores for the conflict, negative expectations and closeness facets of student's relationship quality with teachers over a 1-year time lapse and 1-year follow-up measures of students' achievement and emotional and behavioral difficulties. Students' outcome measures at baseline (t1) and gender were added as covariates in the analyses.

## Results

### Descriptive statistics

Table 1 reports the descriptive statistics computed on the study variables as measured at baseline (T1) and follow-up (T2) in the overall sample, and by gender group. On average, participants showed low levels of emotional symptoms and problematic behaviors, conflict and negative expectations with teachers. In turn, they reported medium levels of prosocial behaviors, closeness with teachers and academic achievement. Paired-sample *t*-tests indicated significant negative transition-related changes in hyperactivity/inattention, prosocial behaviors, the conflict and negative expectations facets of students' relationship quality with teachers, as well a significant increase in academic achievement. Based on widely accepted thresholds for Cohen's *d* (Cohen, 1988), although associated with statistically significant changes, effect sizes for conflict, negative expectations, prosocial behaviors and academic achievement, were only small (*d* > 0.20), while change in hyperactivity/inattention was negligible.

**Table 1:** **A study variable in the overall sample and by gender**.

	**Female**					**Male**					**Overall sample**				**Mean change**	**Cohen's *d***
	**T1**		**T2**		**Normative data**	**T1**		**T2**		**Normative data**	**T1**		**T2**			
	***M***	***SD***	***M***	***SD***	***M (SD)***	***M***	***SD***	***M***	***SD***		***M***	***SD***	***M***	***SD***		
SDQ Emotional symptoms	1.40	2.05	1.29	1.69	1.98 (2.32)	1.88	2.28	1.18	1.54	1.74 (2.07)	1.53	2.02	1.23	1.65	−0.30	0.161
SDQ Conduct problems	1.04	1.43	.98	1.51	1.25 (1.79)	1.85^**^	2.10	1.31	1.84	1.82 (2.00)	1.47	1.79	1.15	1.70	−0.32	0.185
SDQ Hyperactivity/Inattention	2.19	2.12	1.96	1.62	2.65 (2.82)	3.89^**^	2.59	3.21^**^	2.43	3.84 (3.02)	3.08	2.30	2.67	2.16	−0.41^*^	0.184
SDQ Peer relationship problems	1.50	1.59	1.38	1.33	2.03 (2.11)	2.11^*^	1.84	1.71	1.55	2.21 (1.89)	1.68	1.64	1.55	1.49	−0.13	0.084
SDQ Prosocial behaviors	7.60	2.49	6.89	2.03	7.28 (2.52)	6.07^**^	2.91	5.73^*^	2.08	5.86 (2.64)	6.88	2.77	6.26	2.18	−0.62^*^	0.244
SPARTS-Closeness	15.52	6.86	16.62	7.36		15.30	6.47	15.26	6.59		15.61	6.88	15.99	7.03	0.38	−0.055
SPARTS-Conflict	8.68	6.38	5.62	4.70		12.21^**^	8.54	9.53^*^	10.58		10.11	7.41	7.49	8.26	−2.62^**^	0.322
SPART-Negative expectations	8.75	4.37	6.40	4.25		8.43	4.35	6.26	5.56		8.25	4.12	6.41	4.83	−1.84^**^	0.403
Academic achievement	7.30	2.02	7.84	1.60		6.36^**^	2.08	7.24^*^	1.74		7.02	2.02	7.54	1.68	0.52^*^	−0.204

*Normative data were available for SDQ only (Tobia et al., 2011)*.

** p < 0.01

* *p < 0.05*.

A few gender differences emerged. At T1, males reported higher scores on conduct problems, hyperactivity/inattention, peer relationship problems and student-teacher conflict, and lower prosocial behaviors and academic achievement than female students. A similar pattern emerged at T2, with the exception of conduct problems, which showed no difference between the groups.

### Correlation analyses

Table 2 shows the correlations between study measures as assessed at T1. Academic achievement showed negative correlations with SDQ subscales assessing conduct problems, hyperactivity/inattention, and peer relationship problems, and with SPARTS conflict subscale. In turn, positive correlations emerged between academic achievement and both SDQ prosocial behaviors and SPARTS closeness subscales.

**Table 2:** **Correlations among study variables (T1)**.

	**AA**	**ES**	**CP**	**HI**	**PP**	**PS**	**CL**	**CO**
Academic achievement								
SDQ Emotional symptoms	−0.17							
SDQ Conduct problems	−0.53^**^	0.36^**^						
SDQ Hyperactivity/Inattention	−0.68^**^	0.40^**^	0.66^**^					
SDQ Peer relationship problems	−0.28^**^	0.29^**^	0.27^**^	0.27^*^				
SDQ Prosocial behaviors	0.48^**^	−0.07	−0.60^**^	−0.49^**^	−0.40^**^			
SPARTS-Closeness	0.23^*^	0.12	−0.28^**^	−0.26^**^	−0.03	0.27^*^		
SPARTS-Conflict	−0.31^**^	−0.09	0.40^**^	0.35^**^	0.14	−0.42^**^	−0.53^**^	
SPARTS-Negative expectations	−0.18	0.16	0.19	0.19	0.10	−0.15	−0.31^**^	0.52^**^

*Variable labels: AA, Academic achievement; ES, emotional symptoms; CP, conduct problems; HI, hyperactivity/inattention; PP, peer relationship problems; PS, prosocial behaviors; CL, Closeness; CO, Conflict; NE, Negative expectations*.

** p < 0.01

* *p < 0.05*.

SDQ subscales showed many significant inter-correlations: Subscales assessing emotional symptoms, conduct problems, hyperactivity/inattention, and peer relationship problems were all positively inter-correlated, and with the exception of the emotional symptoms subscale, all revealed significant negative correlations with the prosocial behaviors subscale. SPARTS subscales were also significantly inter-correlated: Closeness negatively correlated with conflict and negative expectations, which in turn showed a positive inter-correlation.

SDQ and SPARTS subscales also showed the many significant correlations: SPARTS Closeness subscale negatively correlated with the SDQ subscales assessing conduct problems and hyperactivity/inattention, and positively correlated with prosocial behaviors; the SPARTS conflict subscale showed an opposite correlation pattern, while no correlations emerged between the SPARTS negative expectations subscale and the SDQ subscales.

### Regression analyses

Results of the regressions models, reported in Table 3, indicated transition-related changes in relationship quality between students and teachers (i.e., closeness, negative expectations, and conflict with teachers) as significant predictors of changes in both students' academic achievement and two of the five behavioral dimensions measured by SDQ (conduct problems, hyperactivity/inattention). Specifically, an increase in the level of perceived conflict with teachers significantly predicted an increase in both conduct problems and hyperactivity/inattention symptoms across the two considered time points. Concerning academic achievement, both varying and average levels of closeness with teachers significantly predicted change over time: A perceived increase in closeness in 9th grade, as well as a higher mean closeness level, was associated with an increase in achievement.

**Table 3:** **Regression models: SDQ behavioral dimensions and academic achievement (T2) on student-teacher relationship quality (T1-T2 change and average scores)**.

	**ES**	**CP**	**HI**	**PP**	**PS**	**AA**
Negative expectations (Change)	0.08	0.15	−0.04	0.10	0.08	−0.06
Closeness (Change)	−0.06	0.13	−0.02	−0.04	0.09	0.34^**^
Conflict (Change)	−0.14	0.24^*^	0.33^**^	0.01	0.06	−0.01
Negative expectations (Mean)	0.06	0.15	0.15	0.23	−0.19	0.18
Closeness (Mean)	0.02	−0.06	0.05	−0.06	0.09	0.24^*^
Conflict (Mean)	0.01	−0.00	0.03	0.02	−0.02	−0.03
Gender (1 = Female; 0 = Male)	−0.01	−0.05	−0.14	−0.08	0.24^*^	0.08
T1 score	0.09	0.22^*^	0.42^**^	0.03	0.05	0.11
*R^2^* (*Adj. R^2^*)	0.02 (0.00)	0.23 (0.16)	0.35 (0.29)	0.12 (0.04)	0.14 (0.05)	0.25 (0.18)

*Standardized coefficients are reported. Variable labels: ES, emotional symptoms; CP, conduct problems; HI, hyperactivity/inattention; PP, peer relationship problems; PS, prosocial behaviors; AA, Academic achievement*.

** p < 0.01

* *p < 0.05*.

## Discussion

The first analyses conducted were aimed to identify the behavioral characteristics of the adolescents that took part in the research, as evaluated by their teachers using SDQ. Examination of the normative data (Tobia et al., 2011) did not reveal substantial differences in behavioral outcomes between our sample and the Italian population. The variations recorded in the transition to high school show a small reduction in prosocial behaviors and a significant, but weaker, decrease in hyperactivity/attention. Concerning hyperactivity, the weakness of the transition-related effect may be partly due to normative developmental changes in the executive functions linked with self-regulation, which appear to plateau during in early to mid-adolescence (Ng-Knight et al., 2016), and the relatively short time-span in which observations took place. For the other dimensions examined by the SDQ, instead, there were no significant variations.

Academic achievement reached average scores in third year of middle school and was improved by half a point in the first year of high school. The improvement in academic achievement recorded for the participants of our study is not in line with that reported by literature (Akos and Galassi, 2004; Barber and Olsen, 2004; Benner and Graham, 2009).

Relationship with teachers is perceived by 3rd year middle school pupils as not particularly conflictual, and is characterized by low levels of negative expectations and average levels of closeness in terms of the range of the scales. In the transition to high school, there are variations in the relationship with teachers as perceived by students. In high school, said relationship is marked by lower levels of conflict and negative expectations, while the dimension of closeness shows no significant variation. Therefore, in the transition to high school the quality of the relationship with the teacher, as perceived by the students, is higher. This improvement is not linked to a variation in the level of closeness and sharing with the teacher, but to a reduction in the dimension of conflict and negative expectations. This datum is also in contrast with some of the literature, which reports that high school students tend to describe the relationship with their teachers as being detached, impersonal, oriented to learning and not interested in their individual needs for emotional support or encouragement to be autonomous (Seidman et al., 1996; Barber and Olsen, 2004; Cushman and Rogers, 2008; National Middle School Association, 2010; Scalera and Alivernini, 2010; Eccles and Roeser, 2011).

The regression model underlines the importance of the relationship with teachers as both as a risk or a protection factor, depending on its features. In accordance with the literature, we have found that variations in STR quality affect both academic achievement and some of the students' problem behaviors, namely: conduct problems and Hyperactivity (Lynch and Cicchetti, 1992; Birch and Ladd, 1997; Roeser et al., 1998; Wentzel, 1998; Saft and Pianta, 2001; Henricsson and Rydell, 2004; Ahnert et al., 2006; Murray et al., 2008). More specifically, in the transition we have analyzed, the closeness dimension was linked to the improvement of academic achievement, while the conflict dimension is linked to increases in students' problem behavior.

## Conclusions

The transition to high school is described in the literature as being the most critical, difficult and worrying of all developmental transitions (Southern Regional Education Board, 2002; Barber and Olsen, 2004; Roybal et al., 2014), even though some students report positive feelings and successful integration following their transition to the new school (Zeedyk et al., 2003; NSW Department of Education and Training, 2006; Anderman and Leake, 2007; Turner, 2007; Neild, 2009; Hamm et al., 2010; Rice et al., 2011; Waters et al., 2012). The transition to high school requires special consideration, since it coincides with puberty and with the psychophysical changes that entails and, therefore, can place great pressure on the emotional well-being of adolescents (Akos, 2002; Frey et al., 2009; Neild, 2009). The relationship with teachers plays an important role in this particular development phase by favoring scholastic adaptation and, therefore, affecting the dropout rate, which has been found to rise in the first year of high school. Consequently, given the importance of the educational relationship, the analysis of a student's situation in class should also look closely at the quality of the relationship with teachers, and not just at academic results in individual subjects, since these results too are related to the quality of the relationship. Our results show that both the closeness and the conflict dimensions, as perceived by students, are influential and can affect behavior, individual adaptation in class and academic achievement. Therefore, we hope that future interventions will be designed to improve the quality of the STR at middle and high school, so that said relationship may become a protective factor for students. The STR is one of the main factors that influence the degree to which students feel a bond with their school community, and determines their scholastic well-being (Libbey, 2004; Noddings, 2005; Schussler and Collins, 2006; Nichols, 2008; Suldo et al., 2009). Furthermore, it favors a reduction of problem behaviors and an increase in positive and prosocial attitudes in the classroom (Wentzel, 1994; Garnefski and Diekstra, 1996; Birch and Ladd, 1997; Hughes and Kwok, 2007; Close and Solberg, 2008), as well as fewer absences and a lower risk of dropping out and engaging in criminal activity (Finn, 1993; Blum and Rinehart, 1997; Hamre and Pianta, 2005).

Our study suffers from some limitations. First of all, it suffers from sample mortality and from a non-representative sample: it would be useful to plan new longitudinal research studies, expanding sample size and diversifying the territorial and scholastic settings. For example, this would enable the creation of a study on the possible differences linked to a school's territorial localization (i.e., urban or rural) or to the type of high school attended (lyceum, technical or vocational school, etc.). Subsequently, it would be possible to investigate whether the STR plays the same role in these cases, or whether, in some scholastic or territorial contexts, it may have a different influence on students' adaptation to school and their behavior. Finally, another fruitful line of research could focus exclusively on situations that are more at-risk, by designing interventions to improve STRs in order to assess whether this can reduce student dropout rate.

## Author contributions

CL was involved with the design and interpretation of this work as well as writing and revising the manuscript. LP were involved in the acquisition of the data and discuss it. DM and MS were involved in methodology and analysis of the data.

### Conflict of interest statement

The authors declare that the research was conducted in the absence of any commercial or financial relationships that could be construed as a potential conflict of interest.
